# Description of an Ultrasound-Guided Transverse Approach to the Transversus Thoracis Plane Block and Evaluation of Injectate Spread in Canine Cadavers

**DOI:** 10.3390/ani11092657

**Published:** 2021-09-10

**Authors:** Manuel Alaman, Adrián González-Marrón, Cristina Lorente, Cristina Bonastre, Alicia Laborda

**Affiliations:** 1 Department of Animal Pathology, Faculty of Veterinary Medicine, University of Zaragoza, C/Miguel Servet 177, 50013 Zaragoza, Spain; malaman@unizar.es (M.A.); alaborda@unizar.es (A.L.); 2 Hospital Veterinario Anicura Valencia Sur, Avda. Picassent, 28, 46460 Silla, Spain; cristinalorenteroig@gmail.com; 3 Group of Evaluation of Health Determinants and Health Policies, Department of Basic Sciences, Universitat Internacional de Catalunya, 08159 Barcelona, Spain; agonzalezm@uic.es; 4 Instituto Universitario de Investigación Mixto Agroalimentario de Aragón (IA2), University of Zaragoza, 50013 Zaragoza, Spain

**Keywords:** dog, fascial block, intercostal nerve, mastectomy, regional anaesthesia, sternotomy

## Abstract

**Simple Summary:**

In humans, the aim of the transversus thoracis plane block is to desensitise the intercostal nerves running through this plane, providing analgesia to the anterior chest wall. Our objective was twofold: describing an ultrasound-guided transverse approach to the transversus thoracis plane and evaluating the spread of two injectable volumes in canine cadavers. Gross anatomy of the ventral thoracic area and sonoanatomy between the fifth and sixth costal cartilages were described in two dog cadavers. Eight cadavers were used to describe this approach and were subsequently dissected to evaluate the injectate spread and the intercostal nerves staining after low volume (0.5 mL kg^−1^) and high volume (1 mL kg^−1^) dye-lidocaine injection. After all injections, the injectable solution was distributed along the transversus thoracis plane, staining a median number (range) of 3 (2–4) and 4 (3–5) nerves with low and high volume, respectively (*p* = 0.014). The transverse approach to the transversus thoracis plane is a feasible, single injection point technique that provides the staining of several intercostal nerves. The injection of high versus low volume increases the number of stained nerves.

**Abstract:**

Transversus thoracis plane (TTP) block has demonstrated to produce analgesia in humans undergoing median sternotomy. The objectives of the study were to describe an ultrasound-guided transverse approach to the transversus thoracis plane (t-TTP) and to evaluate the spread of two injectable volumes in canine cadavers. Two cadavers were used to describe relevant gross anatomy of the ventral thoracic area and sonoanatomy between the fifth and sixth costal cartilages. Then, eight cadavers were used to describe the ultrasound-guided injection into the TTP and were dissected to evaluate the injectate spread and the intercostal nerves staining with two different dye-lidocaine volumes: low volume (LV) 0.5 mL kg^−1^ and high volume (HV) 1 mL kg^−1^. To compare the spread between both volumes the Fisher’s exact test and Wilcoxon signed-rank test were used. The solution spread along the TTP after all injections, staining a median number (range) of 3 (2–4) and 4 (3–5) nerves with LV and HV, respectively (*p* = 0.014). The injection of HV versus LV increases the number of stained nerves. Ultrasound-guided t-TTP is a feasible technique that provides staining of several intercostal nerves with a single injection site, so it could be useful to provide analgesia to the ventral chest wall.

## 1. Introduction

Although opioids have traditionally been used to manage perioperative pain produced by median sternotomy, regional anaesthesia has been described as an effective alternative to opioids, decreasing its consumption, side effects, and improving recovery [1,2]. Several anaesthetic blocks have demonstrated to produce analgesia in humans undergoing median sternotomy, including neuraxial techniques, transversus thoracis plane (TTP) block, thoracic paravertebral block, erector spinae plane block, and parasternal block [3].

In veterinary medicine, neuraxial techniques and peripheral anaesthetic blocks, as thoracic paravertebral block, erector spinae plane block, serratus plane block, intercostal blocks or pleural irrigation with local anaesthetics, have been used to provide chest wall analgesia [4,5,6,7,8,9]. No studies have been reported suggesting the efficacy of the TTP block to provide analgesia to the ventral aspect of the chest wall in dogs.

The ultrasound-guided TTP block is performed to inject an anaesthetic solution into the interfascial plane between the transversus thoracis muscle (TT) and the internal intercostal muscles. In humans, the local anaesthetic spread achieves the desensitization of the anterior portion of the intercostal nerves, providing analgesia to the anterior chest wall including the midline skin, sternum, and costal pleura. In human cadaveric studies, a single injection of 15–20 mL of dye solution into the TTP, between the fourth and fifth costal cartilages, achieved the staining of the anterior portion of the intercostal nerves from T1 to T6 [10,11].

The TTP block has been reported as an effective technique to provide analgesia in humans undergoing pericardiocentesis [12] and cardiac surgery via median sternotomy [13,14,15], to relieve chronic pain related to sternal lesions or costochondritis [16,17] and, combined with other fascial plane blocks, to control acute pain in patients undergoing breast surgery [18,19]. Two cases of continuous TTP block through bilaterally placed catheters allowed successful control of perioperative pain associated with median sternotomy [20].

Sagittal and transverse approaches to TTP block have been reported in human literature [21]. The sagittal approach was recently described in veterinary literature [22]; however, to our knowledge, there are no references to the ultrasound-guided transverse approach to the transversus thoracis plane (t-TTP).

Thus, the objectives of this study were: (1) to evaluate the gross and ultrasonographic anatomy of the TTP; (2) to describe the ultrasound-guided transverse approach to the TTP in dog cadavers; (3) to compare the spread of high volume (HV) 1 mL kg^−1^ and low volume (LV) 0.5 mL kg^−1^ of a methylene blue-lidocaine solution and the staining of the intercostal nerves following the t-TTP injection between fifth and sixth costal cartilages.

The hypotheses of this study were: (1) the TTP can be identified ultrasonographically in dog cadavers and the needle tip can be well recognised when the injection is performed using the t-TTP approach; (2) the injection of a methylene blue-lidocaine solution in the TTP through a transverse approach will stain the intercostal nerves responsible for ventral chest wall innervation and; (3) more nerve structures will be stained when administering HV of the injectable solution instead of LV.

## 2. Materials and Methods

### 2.1. Study Design

The study was approved by the Animal Ethics Experimentation Committee of the University of Zaragoza (PI12/21NE). Dogs with sternal congenital malformations and/or penetrating wounds in the thorax or abdomen were excluded. The project enrolled 10 canine cadavers that were euthanized for reasons unrelated to the study. Cadavers were thawed at room temperature for 24–48 h. The thoracolumbar region was clipped in all cadavers.

The study was divided in two phases: during phase I, two cadavers (9.3 and 20.1 kg) were used to describe relevant gross anatomy of the ventral thoracic area and the sonoanatomy between the fifth and sixth costal cartilages. Phase II enrolled eight cadavers weighing a median of 15.6 kg (range 7.8–24 kg), which were used to describe the ultrasound-guided injection of a methylene blue-lidocaine solution into the TTP by a transverse approach and to evaluate the injectate spread and nerve staining. The ultrasound anatomical evaluation and guided injections were performed with a linear ultrasound transducer (14–6 MHz, L14-6Ns, Mindray Bio-Medical Electronics Co., Shenzhen, China) connected to a portable ultrasound machine (Mindray M9 Vet, Mindray Bio-Medical Electronics Co., China) by the same experienced investigators (M.A., C.L.).

### 2.2. Phase I: Anatomical and Ultrasound Study

Each dog was placed in dorsal recumbency with forelimbs oriented cranially and dissection was performed on one hemithorax of each cadaver. Once the skin was removed, the pectoralis superficialis and pectoralis profunda muscles were separated from the sternum and laterally reclined to expose the rectus thoracis and rectus abdominis muscles. These muscles, which were sectioned between the first costal cartilage and caudally to the xiphoid process, were reclined dorsally to identify the distal muscular branches of the intercostal nerves after crossing the internal and external intercostal muscles. The external and internal intercostal muscles between the second and ninth costal cartilages were separated from their caudal costal cartilage insertion and from the sternum and reclined cranially in order to identify the TTP, the internal intercostal membrane, the path of the intercostal nerve and the internal thoracic artery and vein. The transversus thoracis muscle was identified and separated from the endothoracic fascia and the costal pleura.

Then, the contralateral region of the ventral thoracic wall between the fifth and sixth costal cartilages was ultrasonographically evaluated and correlated with the anatomical dissection to identify ultrasonographic references for the injection of TTP. Once the investigators become familiar with the ultrasound anatomy, a 63 mm and 22-gauge spinal needle (BD Spinal Needle; BD Medical, Franklin Lakes, NJ, USA) was used to test a preliminary in-plane approach to TTP, injecting 0.1 mL of methylene blue (methylthioninium chloride injection 1% *w*/*v*; Martindale Pharma, High Wycombe, UK) between the transversus thoracis muscle and the internal intercostal muscle, laterally to the internal thoracic artery and vein. A dissection procedure was repeated in the injection side to confirm the spread of the methylene blue-lidocaine solution into the TTP. Finally, the thoracic wall was sectioned bilaterally and longitudinally to its sagittal midline to expose the dorsal aspect of the ventral thoracic wall and ventral mediastinum.

### 2.3. Phase II: Evaluation of the Injectable Solution Spread

A solution of 200 mL of lidocaine (Lidocaine 2%, B. Braun Melsungen AG, Melsungen, Germany) and 0.2 mL of methylene blue was prepared and 0.5 mL kg^−1^ (LV) and 1 mL kg^−1^ (HV) were loaded in two syringes for each of the eight canine cadavers. One of the two volumes was injected into the TTP in one side of the thorax and the other into the contralateral side, after a randomization performed using Microsoft Excel for Windows Version 2103 (Microsoft Corporation, Redmond, WA, USA). The investigator who performed the anatomical dissections (M.A.) was different from the one that injected the solution (C.L.), being the first one blinded to the injection side randomization.

#### 2.3.1. Ultrasound-Guided t-TTP Injection

**Figure 1:**
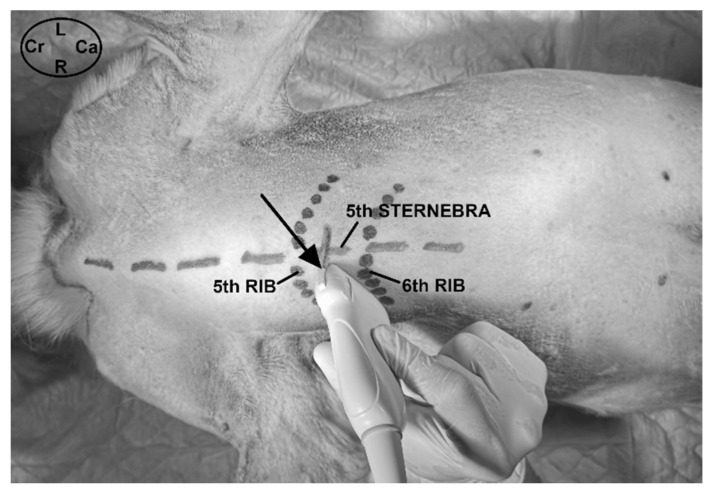
Transverse ultrasound-guided approach to the transversus thoracis plane at the level of the fifth interchondral space in dogs. The transducer was positioned parallel to the fifth and sixth costal cartilages, with the marker located medially and orientated in a caudolateral to craniomedial to direction. The black arrow indicates the direction of the needle in an in-plane technique. Ca, caudal; Cr, cranial; L, left; R, right.

**Figure 2:**
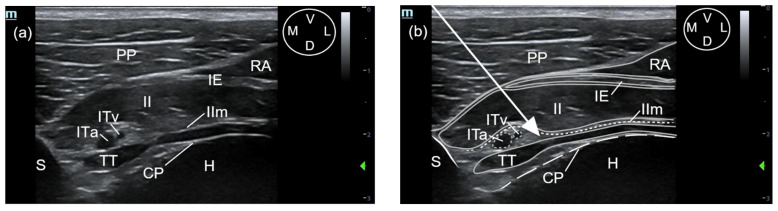
(**a**) Ultrasound image of the fifth interchondral space in a canine cadaver, showing the anatomical structures observed during the transverse approach to the transversus thoracis plane (t-TTP). (**b**) The same ultrasound image with the edges of the muscular structures (solid grey lines), the internal intercostal membrane (short dashed white lines), fifth sternebra (solid white line), vascular structures (short dashed grey lines) and costal pleura (long dashed lines) superimposed to facilitate their identification. The white arrow shows the needle pathway and the needle tip located into the transversus thoracis plane. CP, costal pleura; D, dorsal; H, heart; IE, external intercostal muscle; II, internal intercostal muscle; IIm, internal intercostal membrane; ITa, internal thoracic artery; ITv, internal thoracic vein; L, lateral; M, medial; PP, pectoralis profunda muscle; RA, rectus abdominis muscle; S, Sternum; TT, transversus thoracis muscle; V, ventral.

Each cadaver was positioned in dorsal recumbence with the anterior limbs oriented cranially. The linear ultrasound transducer was positioned in a parasternal position slightly lateral to the fifth sternebra, between the fifth and sixth costal cartilage and parallel to the longitudinal axis of both costal cartilages with the marker oriented medially (Figure 1). The following thoracic structures were identified: pectoralis profunda, rectus abdominis and external and internal intercostal muscles, internal intercostal membrane, fifth sternebra, internal thoracic artery and vein, transversus thoracis muscle and costal pleura (Figure 2).

**Figure 3:**
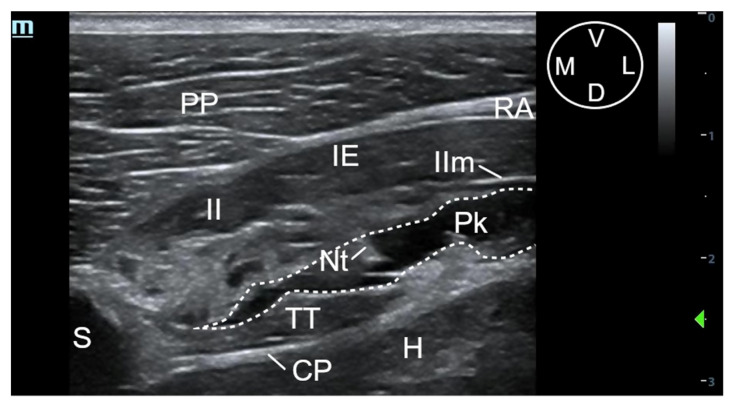
Ultrasound image of the pocket of fluid formation after the injection of a methylene blue-lidocaine solution using the t-TTP approach, at the level of the fifth interchondral space. The pocket of fluid is delimited by the dotted line. CP, costal pleura; D, dorsal; IE, external intercostal muscle; II, internal intercostal muscle; IIm, internal intercostal membrane; L, lateral; M, medial; Nt, needle tip; Pk, pocket of fluid, PP, pectoralis profunda muscle; RA, rectus abdominis muscle; S, sternum; TT, transversus thoracis muscle; V, ventral.

A 63 mm, 22-gauge spinal needle (BD Spinal Needle; BD Medical, Franklin Lakes, NJ, USA) was inserted in a ventromedial-to-dorsolateral direction with an in-plane technique. The needle was advanced through the pectoralis profunda, rectus abdominis, external and internal intercostal muscles, and internal intercostal membrane until the tip was positioned into the TTP, ventral to TT muscle and laterally to the internal thoracic artery and vein (Figure 2 and Figure 3). To confirm the presence of the needle tip into the target plane, a small amount of the injectable solution was slowly administered until a small pocket of fluid was visualized between the internal intercostal membrane and the transversus thoracis muscle. Then, the remaining volume corresponding to that side of the thorax was injected (Figure 3). If the solution injection did not reach the target plane, the tip of the needle was redirected and all the procedure was repeated. In all the injections, the quality of the needle tip ultrasound visualization was evaluated (Appendix A). The procedure was repeated on the contralateral side.

#### 2.3.2. Evaluation of Methylene Blue-Lidocaine Solution

Initially, the dissection of both hemithoraxes was performed following the same protocol described above. Then, the thoracic wall was sectioned bilaterally and longitudinally to its sagittal midline to expose the mediastinum and the dorsal aspect of the ventral chest wall. The first aim of the evaluation of the spread was to identify the number of stained intercostal nerves. A nerve structure was considered stained when all its quadrants were dyed for more than one centimetre in length.

Afterwards, the distribution pattern of the injectable solution was described and recorded in five different planes: (1) between the rectus abdominis and the intercostal muscles (RIP); (2) between the internal intercostal and the transversus thoracis muscles (TTP); (3) between the transversus thoracis muscle, the endothoracic fascia and the costal pleura; (4) through the mediastinum and (5) intrapleural staining.

The TTP was further divided in different segments using each interchondral space as a reference. A segment was considered stained when dye was observed along its total longitudinal axis. The dissection procedure was repeated in the contralateral side.

### 2.4. Statistical Analysis

Qualitative variables were described with absolute frequency and percentage and quantitative variables with median and range.

The spread of methylene blue-lidocaine solution and the staining of the intercostal nerves, along the five planes described above, were compared according to the injected volume (LV or HV). The Fisher’s exact test was used to compare the proportions of stained nerves and planes according to the volume injected. Wilcoxon signed-rank test was used to compare the mean ranks of intercostal nerves and TTP interchondral segments stained with LV and HV methylene blue-lidocaine solution. The level of significance was set at 0.05. Statistical analyses were performed using IBM SPSS statistics version 19.0 for Windows (SPSS Inc., Chicago, IL, USA).

## 3. Results

### 3.1. Phase I: Anatomical and Ultrasound Study

#### 3.1.1. Gross Anatomical Description

The TTP is located between the second and the seventh internal intercostal muscles, and the TT muscle at the ventral region of the thoracic wall. Each internal intercostal muscle inserts at its cranial rib through the internal intercostal membrane. This membrane was identified as a thin layer of connective tissue intimately attached to the medial aspect of these muscles. The fibres of the internal intercostal muscles course cranioventrally from the cranial border of one costal cartilage to the caudal border of the anterior costal cartilage, filling the interchondral space from the costochondral junctions to the lateral aspect of the sternum. Each internal intercostal muscle is located medial to an external intercostal muscle, which extends distally without contacting the sternum in the first nine or ten interchondral spaces. The TT muscle is a flat, fan-shaped muscle, which was identified covering the medial aspect of the second to the eighth costal cartilages. The TT muscular fibres arise from the dorsolateral aspect of the sternum, between the second sternebra and the caudal aspect of the xiphoid process and run laterally to be attached to the medial aspect of the second to the seventh costal cartilages, ventral to the costochondral junction. The medial aspect of the TT muscle is covered by the endothoracic fascia and the costal pleura, which were identified macroscopically as a single and narrow tissue layer. The costal pleura extends over the dorsal aspect of the sternum to connect with the mediastinal pleura forming the costomediastinal recess. The dorsal to the TT muscle, the costal pleura and the endothoracic fascia cover the medial aspect of the internal intercostal muscles. The TTP contains a variable amount of fat and the internal thoracic artery and vein, running parallel to the lateral aspect of the sternum.

Each intercostal nerve, with its corresponding intercostal vein and artery, forms a neurovascular bundle, which runs, along its proximal portion, close to the caudomedial border of its rib and between the costal pleura and the internal intercostal membrane. Subsequently, the T2 to the T7 intercostal nerves run along the TTP and give distal muscular branches that reach the TT muscle and the rectus abdominis muscle getting across the internal intercostal muscles. The distal portion of the intercostal nerves pass through the internal intercostal membrane and continue their path between the fibres of the internal intercostal muscles until they reach the lateral aspect of the sternum, where they become a ventral cutaneous branch that innervates the skin located on the ventral midline of the chest wall. The fifth to the seventh ventral cutaneous branches innervate the medial portion of the thoracic mammary glands.

#### 3.1.2. Description of the Sonoanatomy

The fifth sternebra was visualised as a triangular-shaped structure with a hyperechoic surface and acoustic shadow underneath. The pectoralis profunda muscle was visualised as a thick structure ventral and lateral to the sternum, separated from it and from the intercostal muscles by the rectus abdominis muscle, which appeared as a hypoechoic muscle with a thin and hyperechoic parasternal aponeurosis. The external intercostal muscle covers the lateral aspect of the internal intercostal muscle except for its most distal portion close to the sternum, where contacts laterally with the aponeurosis of the rectus abdominis muscle. The internal intercostal muscle extends distally until the lateral aspect of the sternum, appearing as a thick hypoechoic structure with a thin hyperechoic line on its craniomedial margin, corresponding to the internal intercostal membrane. The TTP is the virtual space between the internal intercostal and the TT muscles. This plane contains a variable amount of hyperechoic fat, mainly located adjacent to the lateral aspect of the sternum, and the internal thoracic artery and vein. These vascular structures appear as two rounded anechoic structures adjacent to the lateral aspect of the sternum. The TT muscle is seen as a hypoechoic structure extending immediately lateral to a bright hyperechoic line corresponding to the costal pleura. A small amount of fat was observed between the pleura and the TT muscle close to the sternum. The heart was visualised immediately dorsal to the costal pleura (Figure 2).

### 3.2. Phase II: Evaluation of the Injectable Solution Spread

#### 3.2.1. Ultrasound-Guided t-TTP Injection

The sonographic landmarks were visualized in all hemithoraxes. The visualisation of the needle tip into the TTP was scored as good in 12 out of 16 injections and poor in 4 out of 16 injections. Fat was easily visualized into the TTP in all injections, facilitating the identification of the correct plane. In all injections, the formation of a pocket of fluid between the TT and internal intercostal membrane could be recognized (Figure 3).

#### 3.2.2. Evaluation of Methylene Blue-Lidocaine Solution

A median of 3 (range 2–4) and 4 intercostal nerves (range 3–5) (*p* = 0.105) were stained using LV and HV, respectively. In six out of the eight dogs, the number of intercostal nerves stained with HV was higher than using LV and in two dogs the number was equal (*p* = 0.014). In all stained intercostal nerves, dye was observed along the portion of the nerve running through the TTP, medially to the internal intercostal membrane, and between the costal pleura and the internal intercostal membrane, proximal to the TTP.

In all injections, dye spread was observed along the longitudinal axis of the TTP staining a median (range) of 4 (3–5) and 5 (4–6) interchondral spaces using LV and HV, respectively (*p* = 0.015). In 2 of 16 injections, intramuscular distribution of the dye in the punctured internal intercostal muscle was observed.

**Figure 4:**
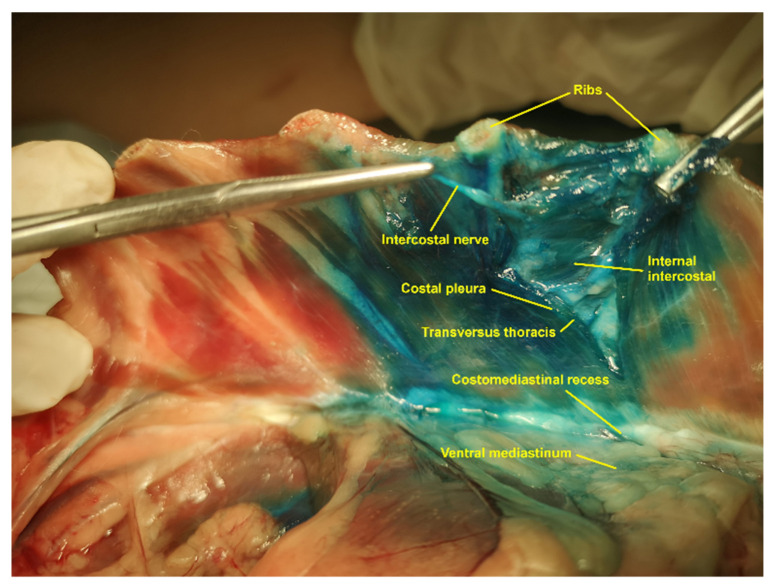
Anatomical dissection of the transversus thoracis plane, dorsal aspect of the ventral thoracic wall and ventral mediastinum. TT muscle was sectioned at the level of the fifth interchondral space to expose the intercostal nerves and the internal intercostal muscle. The image shows the methylene blue-lidocaine solution staining the intercostal nerve, the TTP, the costomediastinal recess and the ventral mediastinum. TT, transversus thoracis; TTP, transversus thoracis plane.

No methylene blue-lidocaine solution was observed into the RIP after any injection. In all cases, methylene blue-lidocaine spread was found medial to the TT muscle, between the costal pleura and the internal intercostal membrane, close to the costochondral junction. After all LV and HV injections, staining was observed between the costal pleura and the TT muscle and into the mediastinum as well (Figure 4). No intrapleural staining was identified in any case.

**Figure 5:**
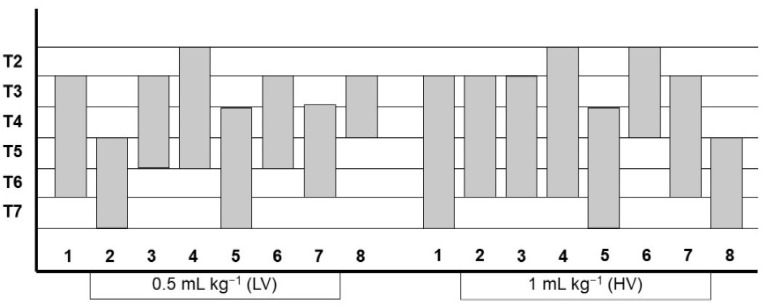
Staining of the T2 to T7 intercostal nerves after the injection of 0.5 mL kg^−1^ (LV) and 1 mL kg^−1^ (HV) of methylene blue-lidocaine solution in eight canine cadavers (a total of 16 injections). Each case is represented by its corresponding number from 1 to 8. HV, high volume; LV, low volume.

All the data referring to the detection of the methylene blue-lidocaine solution in the different evaluated structures are shown in Figure 5 and Table 1 and Table 2.

## 4. Discussion

The present study describes the sonoanatomical landmarks for the t-TTP approach in canine cadavers. This approach enables the administration of a methylene blue-lidocaine solution in the target plane using an in-plane technique and the staining of multiple intercostal nerves between T2 and T7 with a single injection point, although the results observed are not regular and depend on the volume injected.

In dogs, the distal part of the intercostal nerves between T2 and T9 innervate the ventral aspect of the chest wall, including sternum, costal pleura, TT, internal intercostal and rectus abdominis muscles, medial aspect of the thoracic mammary tissue and a cutaneous area, approximately five centimetres wide, adjacent to the thoracic ventromedial line [23]. The current study shows a significantly higher mean rank of dyed intercostal nerves when HV was injected compared to LV injection in the same cadaver, suggesting that increasing the injected volume could be more effective in desensitizing the ventral chest wall. No stained nerves were found caudally to T7 with both volumes, so T8 and T9 nerves might not be desensitized by TTP block with the approach and injectate volumes described. The results suggest that injection of an anaesthetic solution using the t-TTP approach could be effective to provide analgesia for procedures such as median sternotomy, pericardiocentesis or mastectomy, as occurs in humans [12,13,14,18,19]. However, clinical studies would be necessary to confirm these hypotheses.

In both LV and HV groups, the median number of interchondral TTP segments stained was higher than the median number of intercostal nerves stained, so the presence of dye in an interchondral segment of the TTP did not always result in the staining of the corresponding intercostal nerve. This could be related with the fact that the internal intercostal membrane prevents the staining of the intercostal nerve after it has passed through, so nerve staining would be limited to the proximal region of the TTP.

The dye spread observed between the costal pleura and the internal intercostal membrane, proximal to the TTP and close to the costochondral junction, could occur through the endothoracic fascia present between these structures. Similarly, the presence of staining into the mediastinum could be produced through the endothoracic fascia present between the TT muscle and the costal pleura. This fascia penetrates into the mediastinum through the costomediastinal recess. This potential pathway could correlate the staining observed between the pleura costalis and the TT muscle, within the mediastinal cavity as well as between the costal pleura and the internal intercostal membrane after all injections. The endothoracic fascia has already been suggested as a potential pathway for the distribution of an injectable solution into the thoracic paravertebral space during quadratus lumborum plane block [24].

Ueshima and Otake [25] observed a larger spread of the solution when it was injected in the fourth intercostal space compared to when it was administered in the fifth. These authors suggest that the structure of the TT muscle may lead to differences in the spread of the solution.

Recently, Zublena et al. [22] described a sagittal ultrasound-guided parasternal approach to inject a solution between the TT muscle and the internal intercostal muscle. Traditionally in human medicine, parasternal block consists in multiple intercostal blocks just lateral to the sternal border, injecting an anaesthetic solution between the pectoralis profunda and the intercostal muscles [3,16,26]. Although the authors refer to this technique as a parasternal approach, we believe that corresponds to the sagittal TTP approach described in human literature [3,21]. In this study, Zublena et al. [22] evaluated the spread of a dye solution along the TTP and the staining of the intercostal nerves when two different volumes (0.05 mL kg^−1^ and 0.1 mL kg^−1^) were injected. The results suggested its potential utility to desensitize the distal portion of the intercostal nerves, although multiple intercostal injections would be necessary. The study concluded that, although the injectable solution has the potential to spread to multiple interchondral segments along the TTP, the injected volumes might be insufficient to achieve the staining of multiple intercostal nerves with a single injection point. Our results confirm the multi-segmental distribution of the methylene blue-lidocaine solution along the TTP using a transverse approach, and the staining of a greater number of nerves when increasing the injected volume, suggesting that the t-TTP described might decrease the number of injections necessary to desensitize the intercostal nerves involved in ventral chest wall innervation. Conversely, Ueshima and Otake [21] have reported a wider spread of the injectable solution by a sagittal approach than by a transverse approach in a human clinical study.

Bilateral multiple blocks of the intercostal nerves T2 to T9 have traditionally been used to provide somatic analgesia for canine patients undergoing median sternotomy [8]. In a recent canine cadaveric study, the blinded technique has shown an efficacy of only 58.6% staining the target intercostal nerves. This could lead to the failure of the block, incomplete analgesia and increased systemic analgesic requirements. In the same study, an ultrasound-guided intercostal block technique was described in dog cadavers increasing the success rate of staining the targeted intercostal nerves to 91.4% versus the blinded technique [27]. Bilateral ultrasound guided t-TTP block could be an alternative or a complementary technique to multiple bilateral intercostal blocks in dogs undergoing median sternotomy, as it might decrease the number of injection points required to provide analgesia and the time of execution as well. As suggested by Zublena et al. [22], the TTP approach may produce less ventilatory impairment than that produced by multiple intercostal blocks in patients undergoing medial sternotomy, because the muscles affected by the TTP approach do not play an important role in ventilation.

Ueshima and Otake [28] reported a low risk of complications associated with TTP block in humans, with only two cases of infection in 299 patients undergoing this technique. No cases of pneumothorax, hematoma, or vascular puncture were registered. The approach described in the present study allows an accurate positioning of the needle tip into the TTP, with good visualisation of the ultrasound landmarks described during most of the injections. Results suggest that t-TTP could be a safe approach as no dye was observed into the pleural cavity, heart or lung after any injection. Similarly, no puncture of the internal thoracic artery and vein were observed during any of the injections. Nevertheless, the authors suggest the use of colour Doppler during injections in live animals to identify these vascular structures more easily and minimise the risk of puncture. The volumes used warrant that low concentrations of local anaesthetic are used to avoid toxicity.

The use of thawed cadavers is considered by the authors as one of the main limitations of this study, as it could produce differences in the spread of the injected solution compared with its administration in live animals [29]. Moreover, the use of methylene blue as the only dye injected might create confusion when evaluating which structures have been stained with both injectable volumes, mainly the presence of dye into the mediastinum. Although the lidocaine-methylene blue mixture is frequently used in similar cadaveric studies, we consider this to be another limitation of the study because the effects of a methylene blue solution on the physical properties and spread of a local anaesthetic solution are unknown [30,31]. Another limitation could be the ventilatory expansion of the thoracic cage in live animals, which could impair the execution of the approach. Spinal needles were used in this study because economic reasons, although the authors recommend the use of less traumatic needles, such as Tuohy, in live animals to prevent tissue injury.

## 5. Conclusions

This study showed that the ultrasound-guided t-TTP approach is a feasible technique in canine cadavers. The observed results showed the distribution of the injectable solution along the TTP and the staining of multiple intercostal nerves with a single injection point. In addition, increasing the volume of the injectable solution from 0.5 to 1 mL kg^−1^ was associated with a significant increase in the number of the intercostal nerves stained. Although clinical trials would be necessary to evaluate the efficacy of the t-TTP block, the authors suggest that this ultrasound-guided TTP approach is a regional anaesthetic technique that could provide analgesia to the ventral aspect of the chest wall.

## Figures and Tables

**Table 1:** Counts and percentages of intercostal nerves from T2 to T7 stained according to the injectable volume used.

Intercostal Nerves	LV	HV	*p*-Value *
T2	1 (12.5%)	2 (25.0%)	1
T3	5 (62.5%)	6 (75.0%)	1
T4	7 (87.5%)	7 (87.5%)	1
T5	7 (87.5%)	7 (87.5%)	1
T6	4 (50.0%)	7 (87.5%)	0.282
T7	2 (25.0%)	3 (37.5%)	1

* Fisher’s exact test. HV, high volume (1 mL kg^−1^); LV, low volume (0.5 mL kg^−1^).

**Table 2:** Counts and percentages of transversus thoracis plane interchondral segments from T2 to T7 and stained according to the injectable volume used.

TTP Segment	LV	HV	*p*-Value *
TTP (2)	3 (37.5%)	6 (75.0%)	0.315
TTP (3)	6 (75.0%)	7 (87.5%)	1
TTP (4)	7 (87.5%)	8 (100.0%)	1
TTP (5)	8 (100.0%)	8 (100.0%)	NA
TTP (6)	5 (62.5%)	7 (87.5%)	0.569
TTP (7)	2 (25.0%)	4 (50.0%)	0.608

* Fisher’s exact test. HV, high volume (1 mL kg^−1^); LV, low volume (0.5 mL kg^−1^); NA, Not applicable; TTP, transversus thoracis plane.

## Data Availability

Data supporting the reported results can be sent to anyone interested by contacting the corresponding author.

## Appendix A

**Table A1:** Grading ultrasonographic visualization of the needle.

Grade	Description
Good	The shaft and the tip of the needle can be visualized completely.
Poor	The needle tip is only visualized after gentle in- and out-movements or jiggling of the needle.
Absent	The needle tip cannot be recognized.

## References

[1] Mazzeffi M., Khelemsky Y. (2011). Poststernotomy pain: A clinical review. J. Cardiothorac. Vasc. Anest..

[2] Gregory A.J., Grant M.C., Manning M.W., Cheung A.T., Ender J., Sander M., Zarbock A., Stoppe C., Meineri M., Grocott H.P. (2020). Enhanced recovery after cardiac surgery (ERAS Cardiac) recommendations: An important first step—But there is much work to be done. J. Cardiothorac. Vasc. Anesth..

[3] Raj N. (2019). Regional anesthesia for sternotomy and bypass-Beyond the epidural. Paediatr. Anesth..

[4] Thompson S., Johnson J.M. (1991). Analgesia in dogs after intercostal thoracotomy: Comparison of morphine, selective intercostal nerve block, and interpleural regional analgesia with bupivacaine. Vet. Surg..

[5] Flecknell P.A., Kirk A.J., Liles J.H., Hayes P.H., Dark J.H. (1991). Post-operative analgesia following thoracotomy in the dog: An evaluation of the effects of bupivacaine intercostal nerve block and nalbuphine on respiratory function. Lab. Anim..

[6] Pascoe P.J., Dyson D.H. (1993). Analgesia after lateral thoracotomy in dogs. Epidural morphine vs. intercostal bupivacaine. Vet. Surg..

[7] Portela D.A., Campoy L., Otero P.E., Martin-Flores M., Gleed R.D. (2017). Ultrasound-guided thoracic paravertebral injection in dogs: A cadaveric study. Vet. Anaesth. Analg..

[8] Portela D.A., Fuensalida S., Viscasillas J., Verdier N., Otero P.E., Otero P.E., Portela D.A. (2019). Peripheral nerve blocks of the thorax and abdomen. Manual of Small Animal Regional Anesthesia: Illustrated Anatomy for Nerve Stimulation and Ultrasound-Guided Nerve Blocks.

[9] Asorey I., Sambugaro B., Bhalla R.J., Drozdzynska M. (2021). Ultrasound-guided serratus plane block as an effective adjunct to systemic analgesia in four dogs undergoing thoracotomy. Open Vet. J..

[10] Ueshima H., Takeda Y., Ishikawa S., Otake H. (2015). Ultrasound-guided transversus thoracic muscle plane block: A cadaveric study of the spread of injectate. J. Clin. Anesth..

[11] Fujii S., Vissa D., Ganapathy S., Johnson M., Zhou J. (2017). Transversus thoracic muscle plane block on a cadaver with history of coronary artery bypass grafting. Reg. Anesth. Pain Med..

[12] Fujii S., Bairagi R., Roche M., Zhou J.R. (2019). Transversus thoracis muscle plane block. BioMed. Res. Int..

[13] Abdelbaser I.I., Mageed N.A. (2020). Analgesic efficacy of ultrasound guided bilateral transversus thoracis muscle plane block in pediatric cardiac surgery: A randomized, double-blind, controlled study. J. Clin. Anesth..

[14] Zhang Y., Chen S., Gong H., Zhang B. (2020). Efficacy of bilateral transversus thoracis muscle plane block in pediatric patients undergoing open cardiac surgery. J. Cardiothorac. Vasc. Anesth..

[15] Fujii S., Roche M., Jones P.M., Vissa D., Bainbridge D., Zhou J.R. (2019). Transversus thoracis muscle plane block in cardiac surgery: A pilot feasibility study. Reg. Anesth. Pain Med..

[16] Piraccini E., Biondi G., Byrne H., Calli M., Bellantonio D., Musetti G., Maitan S. (2018). Ultrasound guided transversus thoracic plane block, parasternal block and fascial planes hydrodissection for internal mammary post thoracotomy pain syndrome. Eur. J. Pain.

[17] Aydin M.E., Celik M., Celik E.C., Ahiskalioglu E.O., Selvitopi K. (2020). Transversus thoracic muscle plane block for persistent parasternal pain: The Tietze syndrome. J. Clin. Anesth..

[18] Ueshima H., Kimura S., Otake H. (2016). Bilateral breast cancer resection performed under the bilateral transversus thoracic muscle plane block. J. Clin. Anesth..

[19] Ueshima H., Otake H. (2019). A combination of an erector spinae plane block and a transversus thoracic muscle plane block for partial mastectomy. J. Clin. Anesth..

[20] Ueshima H., Otake H. (2017). Continuous transversus thoracic muscle plane block is effective for the median sternotomy. J. Clin. Anesth..

[21] Ueshima H., Otake H. (2017). Comparison of spread of transversus thoracic plane block by sagittal and transverse approach in a clinical setting. J. Clin. Anesth..

[22] Zublena F., Briganti A., De Gennaro C., Corletto F. (2021). Ultrasound-guided parasternal injection in dogs: A cadaver study. Vet. Anaesth. Analg..

[23] Evans H.E., de Lahunta A., Carioto L. (1993). Spinal nerves. Miller’s Anatomy of the Dog.

[24] Dam M., Moriggl B., Hansen C.K., Hoerman R., Bendtsen T.F., Børglum J. (2017). The pathway of injectate spread with the transmuscular quadratus lumborum block: A cadaver study. Anesth. Analg..

[25] Ueshima H., Otake H. (2016). Where is an appropriate injection point for an ultrasound-guided transversus thoracic muscle plane block?. J. Clin. Anesth..

[26] McDonald S.B., Jacobsohn E., Kopacz D.J., Desphande S., Helman J.D., Salinas F., Hall R.A. (2005). Parasternal block and local anesthetic infiltration with levobupivacaine after cardiac surgery with desflurane: The effect on postoperative pain, pulmonary function, and tracheal extubation times. Anest. Analg..

[27] Thomson A.C.S., Portela D.A., Romano M., Otero P.E. (2021). Evaluation of the effect of ultrasound guidance on the accuracy of intercostal nerve injection: A canine cadaveric study. Vet. Anaesth. Analg..

[28] Ueshima H., Otake H. (2017). Ultrasound-guided transversus thoracic muscle plane block: Complication in 299 consecutive cases. J. Clin. Anesth..

[29] Kull K., Baer G.A., Samarütel J., Sand J., Rosenberg P.H. (1997). Distribution of local anesthetic solution in retromediastinal block. Preliminary experimental results. Reg. Anesth..

[30] Campoy L., Martin-Flores M., Looney A.L., Hollis N.E., Ludders J.W., Stewart J., Gleed R.D., Asakawa M. (2008). Distribution of a lidocaine-methylene blue solution staining in brachial plexus, lumbar plexus and sciatic nerve blocks in the dog. Vet. Anaesth. Analg..

[31] Portela D.A., Otero P.E., Briganti A., Romano M., Corletto F., Breghi G. (2013). Femoral nerve block: A novel psoas compartment lateral pre-iliac approach in dog. Vet. Anaesth. Analg..

